# Servant leadership, brand love, and work ethic: important predictors of general health in workers in the education sector

**DOI:** 10.3389/fpsyg.2024.1274965

**Published:** 2024-04-05

**Authors:** Elena Laura-Arias, Miluska Villar-Guevara, Dany Yudet Millones-Liza

**Affiliations:** ^1^UPG de Ciencias Empresariales, Escuela de Posgrado, Universidad Peruana Unión, Lima, Perú; ^2^EP de Administración, Facultad de Ciencias Empresariales, Universidad Peruana Unión, Juliaca, Perú; ^3^EP de Administración, Facultad de Ciencias Empresariales, Universidad Peruana Unión, Lima, Perú

**Keywords:** servant leadership, brand love, work ethic, general health, education sector, Peru

## Abstract

**Background:**

Building a path aimed at the wellbeing of workers in the education sector is the fundamental basis to encourage quality education. To fill the gap in knowledge and address this aspect by understanding the behavior of the study population, it was proposed as with the objective of determining if servant leadership, brand love and work ethic predict the general health in workers.

**Methods:**

A non-probability sampling was applied for convenience. For this purpose, a sample of 509 workers from Peru was submitted to study, who completed a questionnaire consisting of: scale of servant leadership, work ethic, GHQ-12 and brand love. By applying a quantitative method using a structural equation modeling partial least squares approach.

**Results:**

The present study demonstrated that the three constructs (servant leadership, brand love, and work ethic) predict the general health of workers in a positive and significant way, in a sample of Peruvian workers in the education sector. Furthermore, the results suggest that these factors can be used to improve the health of employees in educational institutions in Peru and possibly in other contexts as well.

**Conclusion:**

Given these results and after knowing the solidity of the predictions, the importance of promoting general health in workers in the education sector.

## Introduction

Currently, many companies have seen the great need to predict future changes in the management of human talent, therefore, one of their priority tasks is to redefine traditional roles and responsibilities, which allow increasing the internal health of the organization through the overall employee health (Pino et al., 2020; Wang et al., 2020; Lunde et al., 2022; Tinella et al., 2022; Bezuidenhout et al., 2023); to achieve this end, organizations must have the ability to discover competent people with leadership qualities that promote better results in their work environment, as well as the use of strategies and mechanisms to develop more love (Tijjang et al., 2023), commitment (Grabowski et al., 2019; Tacadena and Muico, 2022; Mitonga-Monga et al., 2023) and ethics (Grabowski et al., 2019; Sakr et al., 2022; Tacadena and Muico, 2022), from employees to your organization.

Today, there are few studies carried out to predict the general health of employees within the workplace (Shi et al., 2022), in a post-COVID context where various jobs are carried out from the homes of employees; and it is here where there is a greater need to evaluate the changes in the general health of workers for the education sector. On the other hand, important scientific evidence shows that the practice of the leadership style of bosses/directors/managers has a significant impact on the health of workers (Sakr et al., 2022; Tinella et al., 2022; McKimm et al., 2023). Although it is true, the transformational leadership is an affective and high-profile leadership style, which is why many institutions disseminate and practice it (Miao and Cao, 2019), however, servant leadership has become one of the most studied, admired, disseminated and useful in various business sectors, due to the variety of benefits it produces (Kaltiainen and Hakanen, 2022). In recent years, researchers have given greater consideration to the study of servant leadership, and how this construct behaves in the various dimensions of the organization (der Kinderen et al., 2020), since not only favors collaborators, managers, senior managers, but its sphere of influence reaches all stakeholders (Newman et al., 2017; Iqbal et al., 2020; Meuser and Smallfield, 2023).

Taking into consideration the current challenges in the educational environment, where the general health of academic staff is vital for the achievement of the purposes that it pursues, allowing anticipation of the changes and challenges posed in this very relevant field of life itself, it is important consider the factors that have been the reason for this study. Therefore, servant leadership is one that is recognized as a style of leadership focused on serving its followers and satisfying their needs, which in turn produces a shared spirit of purpose, trust, commitment, desire for wisdom, and effort in the community organization (Gocen and Sen, 2021). For another on, brand love is an important factor in the recognition of institutional identity, it is the positive emotional connection with a brand, coming to manifest their love for it, allowing them to be more likely to commit and be loyal to it (Larregui-Candelaria et al., 2019). And in turn, studies on work ethics have been validated in various business sectors, considering it as a set of norms and values that serve as a guideline for the activities of a job, taking the contribution of Sharma and Rai (2015), who carried out the operationalization of this variable in three parameters from the point of view of the collaborator; work as a central interest in life, moral attitude toward work and intrinsic work motivation.

And in this context, general health has an important meaning in the lives of workers in the education sector, where this construct has currently been linked to many others: such as coping strategies (Tinella et al., 2022), depression (Gladstone et al., 2018), job insecurity (Setati et al., 2015), psychological discomfort (Jakubiec et al., 2014), machine learning (Hoekstra et al., 2023), working capacity (Kisiel et al., 2023), quality of life (Bezuidenhout et al., 2023), among other. Understanding general health as the mental, emotional and physical wellbeing that allows us to face challenges (Lunde et al., 2022) and acquire greater concentration in the activities that are carried out (Chavez-Espinoza et al., 2022).

Previous studies have concentrated on some elements as predictors of general health (Ebert et al., 2002; Mirsaleh et al., 2011; Nadi et al., 2020; Malakoutikhah et al., 2022), however, there is still a need for a scientific precedent where the usefulness of general health as a contextual factor to explore the behavior between servant leadership, brand love, and work ethic of educational workers (Dahleez and Aboramadan, 2022).

In that sense, after a diligent review of the aforementioned background, there has been a growing interest in continuing to study these topics, both on the part of academics and professionals in the business and health sectors. Although scientific evidence validates that among the study topics, the one that has caused the greatest interest is general health focused on various contexts. On the other hand, bibliometric indicators reveal the 10 countries that most disseminate their scientific results on these topics, among which are: United States (Meriac et al., 2023), United Kingdom (Wang W. et al., 2022), Iran (Malakoutikhah et al., 2022), China (Yuan et al., 2020), Australia (Sajtos et al., 2021), India (Sharma and Rai, 2015), Pakistan (Iqbal et al., 2020), Netherlands (Fernstrand et al., 2017), Spain (Ruiz-Palomino et al., 2021), and Germany (Moll and Kretzschmar, 2017). The same ones who have applied their study to various areas, sectors and populations, such as: medicine (Ebert et al., 2002), business (Suryani et al., 2022), social sciences (Mustafa et al., 2022), psychology (Jakubiec et al., 2014), economics (Peng et al., 2022), humanities (Raja et al., 2020), among others. When discerning scientific dissemination by country, it has been found that the studies carried out in the Peruvian population (Caycho-Rodríguez et al., 2020; Pino et al., 2020; Chavez-Espinoza et al., 2022; Rocha-Vallejos et al., 2022; Alipio et al., 2023) are very limited, that is, there is very little scientific literature that can provide support and guidance for future studies, and that can provide relevant information to develop communication strategies, health promotion, improving wellbeing and general health in the Peruvian context. Given the prevalence of diseases and the current situation of occupational health, this research aims to fill the knowledge gap and provide a valuable contribution to the academic community and professionals of the sectors involved. Based on existing evidence, the objective was determining whether servant leadership, brand love, and work ethics predict the general health of educational workers.

### Literature review

#### Servant leadership

Among the leadership approaches that focus on the collaborator is the transformational leadership approach that seeks to influence those they lead in order to achieve business objectives (Charbonnier-Voirin et al., 2010; Miao and Cao, 2019; Purwanto, 2020; Sjamsoeddin et al., 2023), unlike the servant leadership approach that is oriented to the wellbeing of those led (van Dierendonck and Nuijten, 2011). In this sense, a prudent review of the literature on this last topic will refer to the founder of the servant leadership movement, Robert Kiefner Greenleaf, a notable researcher who developed the theoretical foundation of this construct and published his famous work in 1970 called, “The Servant as Leader,” where he describes servant leaders as those who lead through service, but always with the aim of satisfying the needs of collaborators, providing them with learning opportunities and improving their self-management skills (Eva et al., 2019); equally helps and guides the group by showing compassion, healing, awareness, persuasion, management, and commitment to its growth (Wang W. et al., 2022).

Likewise, it encourages employees to reveal their doubts, take on challenges and reward this attitude by providing resources to feel this freedom (Page and Wong, 2000; Olesia et al., 2014; Gandolfi and Stone, 2018; Lusiani et al., 2020; Pino et al., 2020; Gocen and Sen, 2021). Recent studies provide new evidence on how organizations, through servant leadership behaviors, help employees not only perform better in their tasks, but also to optimally manage work stress (Quick and Henderson, 2016; Zetterberg et al., 2023), to reduce job exhaustion that is therefore associated with the psychological and physiological health of the employee (Kaltiainen and Hakanen, 2022).

#### Brand love

Approximately since 1990, the relationship between the company and its brand has been investigated (Batra et al., 2012; Bagozzi et al., 2017; Na et al., 2023), in terms of loyalty, trust and commitment toward organizations, for this reason the study highlights the theme concerning the relationship and emotional connection that is established through trust, commitment, and loyalty (Larregui-Candelaria et al., 2019). In the research by Shi et al. (2022), refers that the positive connection between trust in the brand and love for the brand has been recently established. In the same way, brand trust is closely linked to attitudinal loyalty due to its ability to provoke positive emotions in customers and workers, in addition to developing a sense of belonging and commitment. There is also a relationship between the company and the employee, which means that employees can commit themselves and be loyal to the brand of the company where they work, considering the few studies that address the issue, some of them refer to the connection between the commitment of staff that goes beyond performing their professional tasks (Wang and Binti, 2023), is also translated as that emotional connection that employees have with the company and how this can influence their health and wellbeing.

#### Work ethic

The work ethic is considered a construct that indicates how much a person values work in their life. It began as the Protestant work ethic, being a classic work by Max Weber around 1958, being the subject of many studies and discussions for being considered a religiously oriented work, however, over time it was called general ethics from work (Sharma and Rai, 2015). Work ethic is the set of attitudes and behaviors at work, as a motivational construction reflected in behavior (Sakr et al., 2022). In addition, in recent years, a number of investigations show that ethics is a predictor of important results at work, some theorists associate its dimensions with the individual’s job performance (Woehr et al., 2023), job satisfaction and stress (Meriac et al., 2023), therefore, it could be shown that it is somehow related to the health of the worker. Work ethic focuses on how ethical practices in the workplace can improve the health and wellbeing of employees. Recent studies highlight the need to support workers in maintaining ethical behavior in their workplace, even from other non-work settings (Alfano, 2022).

#### General health

World Health Organization (2004) in its definition of health, it includes the three most important dimensions of life, referring to a complete state of physical, mental, and social wellbeing, and not only the absence of conditions or diseases. The definition of general health encompasses a series of components that define it as a continuous process of satisfaction that enables people to develop their abilities and potential naturally (Chavez-Espinoza et al., 2022). General health reflects a person’s perception of physical symptoms, sleep disorders, anxiety symptoms, depressive symptoms, and social functioning (Tinella et al., 2022), reflecting both positive and negative aspects of health (Nadi et al., 2020).

Furthermore, previous studies have shown that general health reflects not only physical health but also mental health (Bezuidenhout et al., 2023), because general health is a broader outcome than physical and mental health (Shi et al., 2022). A wide range of factors are known to be associated with health status (Hoekstra et al., 2023), such as lifestyle (Isola, 2020), socioeconomic status (Nadi et al., 2020), wellbeing at work (de Ceballos and Santos, 2015), anxiety (Malakoutikhah et al., 2022), perceived disease burden (Nielsen et al., 2015), sociodemographic factors (Tinella et al., 2022), work facilitation (Shi et al., 2022), and work ability (Kisiel et al., 2023). By conducting a thorough review, studies show other multidimensional models, however, it is believed that for this non-clinical study it is better suited using the GHQ-12 (Montazeri et al., 2003; Hystad and Johnsen, 2020; Mayhew et al., 2021; Lütke et al., 2022).

### Hypothesis development

#### Servant leadership predicts general health

Servant leadership has emerged as an alternative to mitigate some of the stress experienced by professionals in their fields of work; in this context, the application of the PERMA model is proposed, a model that establishes the way in which each individual chooses to carry out activities that make them happy, contributing these activities to generate a feeling of wellbeing, this feeling being what allows the individual to have a positive experience (Turner, 2022). The service leader brings together certain qualities of honesty, righteousness and selflessness that make his collaborators feel good, as they are immersed in an atmosphere of harmony, thus opening the way to a greater likelihood of greater wellbeing for both the collaborators and the leader (Siu et al., 2015; Zeng et al., 2022). And servant leadership, beyond being an admirable quality, has a special recognition for fostering positive behavioral outcomes and active participation that contributes to the wellbeing of workers (Yuan et al., 2020).

From another perspective (Dollard and Bakker, 2010; Montano et al., 2017), the role of the leader is important for this purpose; thus, the leadership of someone who chooses to give priority to others is required, someone who supports his followers to develop their maximum potential, thus contributing to the mental and psychological health of the workers; that is, in this scenario, the active participation of a service leader who contributes, by his way of acting and proceeding, to the wellbeing of the workers is required; this is a key point in the construction of a healthy work environment (Cottey and McKimm, 2019). This context reinforces the idea that a leader can develop a proactive approach to employee health, since his function, beyond managing human resources, is also based on generating a long-term vision that recognizes how important employee health is to the sustainable success of the company. Based on the above, the following study hypothesis is proposed:

*H1: Servant leadership predicts general health* in workers in the education sector.

#### Brand love predicts general health

The researchers (Ahuvia et al., 2022) establish that a consumer who has a deep affinity for a product or service significantly values the brand, this fact denotes interpersonal connections that transcends or goes beyond the commercial transaction and according to the literature a brand has the ability to create a strong lasting emotional bond that impacts the consumer’s overall wellbeing. Furthermore, considering that brand love is associated with the consumer experience, scholars are convinced that from a holistic approach, brand love independently to support any marketing outcome, can also be part of the positive impact on the overall health of individuals and is that according to evidence, when a consumer develops brand love, it tends to create feelings of belonging and identity, thus releasing a positive impact on the welfare of consumers, their mental and physical health (Junaid et al., 2020; Attiq et al., 2022; Rodrigues et al., 2022); in the context of this study, it is specified that a worker who loves a certain brand in the educational sector, can experience positive feelings of motivation and satisfaction that can boost their contribution to the objectives of the brand, thus maintaining a positive feeling of general health and increase the feeling of commitment to their educational work. In this way, a special emphasis is made that establishes that the investigations that study consumer behavior have begun to focus on consumer welfare that extends to the health of the same, as it is addressed, in some way, that emotional and psychological needs can be addressed within the environment where the brand and the consumer interact (Bairrada et al., 2019). Based on the above, the following study hypothesis is proposed:

*H2: Brand love predicts general health* in workers in the education sector.

#### Work ethic predicts general health

Work ethic has been seen as a positive resource that fosters a sense of psychological wellbeing and general health in workers (Maaz and Farroq, 2017; Raja et al., 2020). From this concept lies how important it is to preserve a positive state of those who make up an organization, thus having a vision of work ethics that extends to form part of the sustainable development of a company and when workers perceive that work ethics are valued within an organization, healthy working environments are promoted (Chukwuma et al., 2023). In this context, special emphasis is given to human resource management systems where, independently of constantly striving for greater competitiveness, it can also improve work ethics practices (Tadesse Bogale and Ayenew Birbirsa, 2023), since according to Bazzy (2018), work ethics allows to break the paradigm that establishes that hard work, effort, and other sacrifices are indispensable to achieve success, this is how work ethics fulfills an important function of interceding for the wellbeing and health of workers, maintaining a healthy balance between the work and personal life of the worker. Based on the above, the following study hypothesis is proposed:

*H3: Work ethic predicts general health*.

## Aims

**FIGURE 1:**
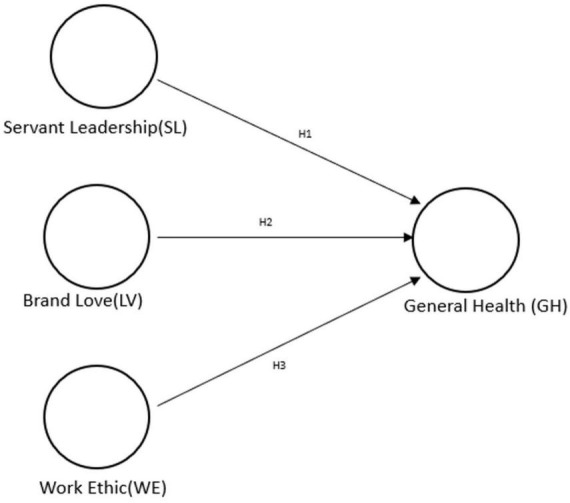
Theoretical model proposed. SL, servant leadership; WE, work ethic; LV, brand love; GH, general health.

This research has focused on determining whether servant leadership, brand love, and work ethics predict the general health of educational workers, and in this way provide relevant information to interest entities that seek to have employees who enjoy greater wellbeing in their work environments; thus, awakening a greater need in leaders to design strategies that seek a healthy balance in their institutions (Figure 1).

## Materials and methods

### Design, procedure, and participants

Cross-sectional explanatory study (Ato et al., 2013).

In the survey, participation was voluntary for the sample of workers from regular basic education (56%), institutes (5%), and universities (40%). The sample comes from Peru. A non-probability sampling was applied for convenience. To be included in this study, participants had to meet the following inclusion criteria: work as a teacher or administrator in the public sector or private from a regular basic institution, institute, or university, have a minimum of 6 months working in the institution regardless of the type of work. Those who did not meet the inclusion criteria were excluded. The data was collected through an online survey platform (Google Forms). A total of 509 questionnaires were considered. From the descriptive analysis of the demographic information (Table 1), 223 were men and 286 women, 43% of the respondents were between 31 and 43 years old and the majority of the subjects (55%) were married, and 36% of them were single, whilst the rest of the participants stated that they were cohabiting, divorced, and widowed. Workers from the coast and mountains predominate (39% each region). The greatest instruction that stood out was higher education (85%). The public sector stands out with 79%. Most of the participants declared less than 14 years of service at their institution (70%). The study was approved by the Ethics Committee of the EPG of the Universidad Peruana Unión (2022-CE-EPG-0000167). Informed consent and assent were obtained from the institutions participating in the study.

**TABLE 1:** Sociodemographic characteristics of the sample.

Demographic information	Frequency (*n*)	Percentage (%)
**Sex**		
Female	286	56.2
Male	223	43.8
**Age range**		
18–30 years	129	25.3
31–43 years	219	43.0
44–56 years	128	25.1
?57 years	33	6.5
**Region of origin**		
Coast	199	39.1
Mountain range	198	38.9
Jungle	103	20.2
Foreign	9	1.8
**Laboral sector**		
Private	401	78.8
Public	108	21.2
**Degree of higher education**		
Primary	5	1.0
Secondary	13	2.6
Technical superior	60	11.8
University higher	431	84.7
**Civil status**		
Married	282	55.4
Cohabitant	21	4.1
Divorced	15	2.9
Single	183	36.0
Widower	8	1.6
**Educational level where you currently work**		
Regular basic education (school/college)	283	55.6
Institute	23	4.5
University	203	39.9
**Years of service**		
0–14	358	70.3
15–29	120	23.6
30–44	28	5.5
45–59	3	0.6

On the other hand, the surveys were self-administered by each participant. Regarding the servant leadership and work ethic scale, its original language was English, and it went through the pretest process. The survey techniques were in English, so they were translated by two native specialists who speak English and Spanish. Likewise, for the application of the four scales (servant leadership, work ethic, general health, and brand love), validation was carried out by expert judgment (six teachers with a master’s degree and doctorate) who evaluated clarity, objectivity, topicality, organization, sufficiency, intentionality, consistency, coherence, methodology, and relevance, resulting in an Aiken’s V indicator of 90.5%, 91.6%, 90.9%, and 90.7%, respectively. Additionally, a focus group session was held which allowed for sematic modifications of the work context, made up of workers from the education sector: two from regular basic education, two from institutes, and two from university. The questionnaire is ready to be applied to these three groups of educational workers. Regarding data collection, the questionnaire was hosted in the Google Form application, which was shared via WhatsApp, Messenger, and Instagram in order to have a greater reach to the study population. Its application lasted 31 weeks, from October 26, 2022 to June 4, 2023.

### Outcome measures

For the purposes of this study, it was estimated to use four scales collected from articles housed in high-impact journals. The data collection instrument was built considering the four scales of the study, finally adding a section of questions to identify the educational level where the participant worked, as well as the institutional region, the labor sector, and other sociodemographic questions. The questionnaire consisted of 32 items: the first 7 items on leadership servant of Gocen and Sen (2021). For work ethics, the scale of Sharma and Rai (2015) that used 10 items. For general health, the scale known as GHQ-12 applied to the Peruvian context by Chavez-Espinoza et al. (2022), this instrument in question was adapted taking into account the questions that were adequate for the realization of the present study, of the 12 items only 6 factorially adequate were used. On the other hand, these first three variables were valued on a Likert-type response scale from 1 to 5, where 1 is “Strongly disagree” and 5 is “Strongly agree.” However, to measure love for the brand, 3 items established by Larregui-Candelaria et al. (2019), the latter was assessed in a 5-point Likert-type response format, ranging from 1: “Completely disagree” to 5: “Completely agree.” Unlike the original instruments, these have been translated and adapted to the Peruvian educational context, considering the particularity of each sector (public and private).

### Data analysis

In the data analysis, the partial least square structural equation modeling (PLS-SEM) was used to test the hypotheses. The PLS-SEM is a comprehensive approach to multivariate statistical analysis that includes measurement and structural components to simultaneously examine the relationships between each of the variables in a conceptual model, which has the characteristic of multivariate analysis, that is, it involves a quantity of variables ≥3 (Hair et al., 2010). In addition, the PLS-SEM was used in the present study because it facilitates the construction of theory (Hair et al., 2011). WarpPLS (Version 8.0) was used to perform the PLS-SEM analysis. This software was used because according to Kock (2014), the WarpPLS provides options to use different algorithms for the external and internal models in the calculation of the scores of the latent variables, such as the path coefficient and the parameters associated with the *p*-value, identifying and taking into account non-linear relationships in the model structural (Kock, 2011).

## Results

The evaluation of a model using PLS-SEM is a two-step process that involves the evaluation of the measurement and structural models (Chin, 2010; Hair et al., 2011; Magno et al., 2022; Mustafa et al., 2022; Peng et al., 2022; Zada M. et al., 2022; Zada S. et al., 2022; Guenther et al., 2023).

### Evaluation of the measurement model

To assess the quality of the reflective constructs, the convergent validity and reliability of the construct must be assessed, that is, internal consistency (Chin, 2010; Hair et al., 2011; Kock, 2015). And the following indicators must be met (Table 2):

**TABLE 2:** Indicators to assess convergent validity and reliability of the constructs.

Indicator	Level
Loading (L)	>0.7
The composite reliability (CR)	>0.7
Cronbach’s alpha (α)	>0.7
The mean-variance extracted (AVE)	>0.5
Variance inflation factor (VIF)	<5
Significance level (*p*-value)	<0.05

Table 3 shows that all the indicators are met. All loadings comply with being greater than 0.7 except for the items SL7 and WE7 whose values are 0.694 and 0.646, respectively, nevertheless, these items have been retained because the reliability indicators as a whole represent a good indicator; the Cronbach’s alpha and CR are greater than 0.7. Likewise, AVE also complies since they are all greater than 0.5. Also the full collinearity VIFs complies since all the values are less than 2.351 which is in the required range; under these terms (Conforti et al., 2014) establish that tolerance lower than 0.2 or an indicator higher than 5 of VIFs represents a multicollinearity problem, for the case of this study the values oscillate between 1.116 and 2.351; this means that, the dispersion of the variables does not have a high correlation between them, this represents a high robustness in the results and estimated coefficient. According to the skewness and kurtosis, it is observed that the data do not have a normal distribution, however, one of the characteristics of the PLS-SEM approach is that it is relatively robust to deviations from normality (Ramírez et al., 2014)and requires assumptions less demanding about the distribution of data (Hair et al., 2013). Since all the indicators comply, we proceed to the discriminant assessment.

**TABLE 3:** Results of the evaluation of the measurement model.

Item	Loading	Skewness	Kurtosis	CR	α	AVE	VIFs
SL1	0.831***	−1.252	1.161	0.938	0.923	0.686	1.841
SL2	0.862***	−0.861	0.150				
SL3	0.850***	−0.776	−0.296				
SL4	0.881***	−1.149	0.865				
SL5	0.815***	−0.491	−0.486				
SL6	0.851***	−1.046	0.582				
SL7	0.694***	−0.665	−0.374				
WE1	0.867***	−1.587	2.035	0.963	0.957	0.724	2.351
WE2	0.843***	−1.447	1.701				
WE3	0.899***	−1.745	2.954				
WE4	0.875***	−1.418	1.880				
WE5	0.817***	−1.326	1.438				
WE6	0.867***	−1.718	2.826				
WE7	0.646***	−0.922	0.025				
WE8	0.864***	−1.613	2.393				
WE9	0.909***	−1.924	3.585				
WE10	0.890***	−1.703	2.974				
LV1	0.900***	−1.659	3.510	0.910	0.851	0.771	1.116
LV2	0.883***	−1.672	3.542				
LV3	0.850***	−1.859	4.225				
GH1	0.764***	−1.096	0.516	0.965	0.955	0.820	1.697
GH2	0.911***	−1.291	1.389				
GH3	0.934***	−1.281	1.292				
GH4	0.931***	−1.357	1.514				
GH5	0.948***	−1.490	1.984				
GH6	0.933***	−1.533	1.871				

The factor loading of items SL7 and WE7 were accepted by decimal approximation. Cronbach’s alpha (α), full collinearity VIFs, ***p < 0.001 (significance level).

Discriminant validity provides an indication of the extent to which each construct is different from other constructs in the model (Chin, 2010). To meet discriminant validity, the square root of the AVE for each construct must be greater than the highest correlation between the construct and other constructs in the model (Chin, 2010; Hair et al., 2011; Kock, 2014). Table 4 shows that the square root of the AVEs for all the constructs is greater than the correlation with the other constructs, indicating that the model has acceptable discriminant validity.

**TABLE 4:** Discriminant validity.

	SL	LV	WE	GH
SL	0.828			
LV	0.272	0.878		
WE	0.667	0.291	0.851	
GH	0.481	0.261	0.632	0.906

SL, servant leadership; WE, work ethics; LV, brand love; GH, general health.

### Goodness of fit of the structural model

Evaluating the fit of the statistical model to the study data involves evaluating the goodness of fit of the structural model. Table 5 shows the six goodness of fit indices that have been considered (Kock, 2014), with a confidence level of 95%. In the case of the present study, the six fit indices suggested that the model fit was more than acceptable. The predictive validity of a construct can be confirmed when the value of its associated *R*^2^ coefficient is greater than zero. This was the case for all values of the endogenous variables in the model, suggesting acceptable predictive validity across the model.

**TABLE 5:** Model fit and quality indices.

Model fit measures and quality indices	Value	Result	Adjustment criteria
Average path coefficient	APC = 0.247; *p* < 0.001	Significant	*p* < 0.05
Average *R*-squared	ARS = 0.429; *p* < 0.001	Significant	*p* < 0.05
Adjusted mean *R*- square	AARS = 0.426; *p* < 0.001	Significant	*p* < 0.05
VIF average block	AVIF = 2.045	Ideally	Acceptable ≤ 5, ideally ≤ 3.3
Average complete collinearity	AFVIF = 1.751	Ideally	Acceptable ≤ 5, ideally ≤ 3.3
Tenenhaus GoF	GoF = 0.567	Big	Small ≥ 0.1, medium ≥ 0.25, large ≥ 0.36

### Structural model evaluation

To evaluate the structural model, two preliminary criteria must be verified and reported: the importance of the path coefficients and the coefficient value of *R*^2^ for endogenous constructs. Each hypothesis is associated with a causal link in the structural model, which represents the relationships between a pair of constructs. Path coefficients have been calculated for each relationship in the model, as well as their corresponding *p*-values. Although the path coefficients must be significant, the value of the *R*^2^ coefficient is highly dependent on the research area. Chin (1998) suggests values of 0.67, 0.33, and 0.19 as, respectively, substantial, moderate, and weak measures of *R*. In behavioral studies, a value of 0.2 for *R*^2^ is generally considered acceptable (Kock, 2013; Hair et al., 2014).

**FIGURE 2:**
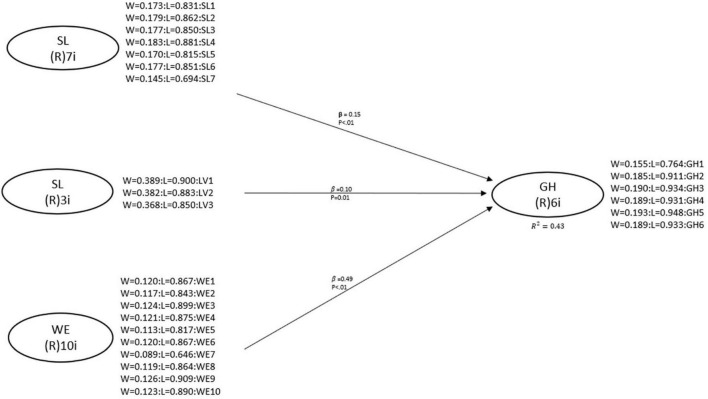
Results of the structural model.

In the present study, the *R*^2^ coefficient for GH was 0.43; this means that the proposed model explains 43% of the observed variability (James et al., 2023); that is, servant leadership, love of the brand and work ethic predict the general health of workers by 43%. Therefore, this value has a high and acceptable level. Table 6 and Figure 2 show the results of the hypothesis tests and the evaluation of the path coefficients. The results show a positive and significant predictive value of SL on GH (H1), of LV on GH (H2), and WE on GH (H3).

**TABLE 6:** Hypothesis test results.

	Hypothesis	Pat coefficient	*p*-Value	Decision
H1	SL–GH	0.151	<0.001	Accepted
H2	LV–GH	0.097	0.014	Accepted
H3	WE–GH	0.492	<0.001	Accepted

For the fit index of the global model, the six indices of goodness of fit have been considered (Kock, 2014), with a confidence level of 95%, the efficiency indices are the following:

• Average Path Coefficient (APC) and *p* < 0.05
• Average *R*-squared (ARS) and *p* < 0.05
• Adjusted mean *R*-square (AARS) > 0.02 and *p* > 0.05
• Average block VIF (AVIF), acceptable if ≤5, ideally ≤3.3
• Average complete collinearity (AFVIF), acceptable if ≤5, ideally ≤3.3
• Tenenhaus GoF, small ≥0.1, medium ≥0.25, large ≥0.36

In the case of the present study, the six fit indices suggested that the model fit was more than acceptable: APC = 0.247, *p* < 0.001; ARS = 0.429, *p* < 0.001; AARS = 0.426, *p* < 0.001; AVIF = 2,045 (acceptable if ≤5, ideally ≤3.3); AFVIF = 1.751 (acceptable if ≤5, ideally ≤3.3); and Tenenhaus GoF = 0.567 (small ≥0.1, medium ≥0.25, large ≥0.36). The predictive validity of a construct can be confirmed when the value of its associated to the coefficient *R*^2^ is greater than zero. This was the case for all values of the endogenous variables in the model, which suggests an acceptable predictive validity in the whole model. This means that the fit indices submitted for evaluation support the robustness of the model, which affirms that servant leadership predicts general health by 15%; likewise, love for the brand also predicts general health by 10%, while work ethic predicts it by 49%; thus, it stands out that work ethic is a strong predictor of the variability of workers’ general health, this information provides an accurate picture to have a better understanding of the factors that predict general health.

## Discussion

The general health of workers has become an innate need in the work environment, especially in the educational sector, where teaching and administrative staff play a fundamental role in the development of students. Thus, it is important to promote the general health of workers in the education sector, since this act not only has benefits for them individually, but also has a positive impact on the work environment and on the quality of the education provided; in this context, this study proposed as its first objective to determine if servant leadership predicts the general health of employees. According to the findings, servant leadership predicts the general health of employees. This statement is supported by various scientists (Hoch et al., 2016; Wang W. et al., 2022; Meuser and Smallfield, 2023), servant leadership has increased significantly among business leaders as it is considered a positive quality that is aimed at making them a role model due to their ethical behavior that contributes to the wellbeing of people; about, Wang Z. et al. (2022) and Iqbal et al. (2020) they show that the servant leader has a high capacity to promote the general health of employees through work motivation and empowerment; In this way, it is argued that promoting servant leadership is an assertive decision that opens the fulfillment of common objectives, thus generating a positive state in workers (Xanthopoulou et al., 2012; Miao and Cao, 2019).

Additionally, a significant index of the brand love has been detected as a predictor of the general health of employees, this behavior is very recurrent, according to Junaid et al. (2020) the east arises as a result of positive feelings and satisfaction in different areas of life. About, Attiq et al. (2022) they highlight that the happiness, attachment and good relationship of people in the same environment can predict general health; specifically in the case of workers in the education sector, Strauss and Daniels (2013) establish that because teachers are exposed to extreme pressures due to daily work with students, parents and educational policies, their general wellbeing is at risk, attributing this fact to unhappiness in the workplace. work, for which they suggest healthy working conditions, which supposes a positive feeling in the school–teacher relationship, this statement is consistent with Li and Miao (2022) and Li et al. (2019), who specify that positive emotions are vital issues in the teaching exercise, so the general health of workers is the result of their attachment or love that they maintain with their institution. And it is that every prosperous social connection established over time generates a special feeling called love, the same one that has the capacity to generate a feeling of wellbeing (Oravecz et al., 2020).

Another of the findings guarantees the prediction of work ethics toward the general health of employees; about, Suryani et al. (2022) they place special emphasis on workers developing positive attitudes, including work ethic, which generates a feeling of wellbeing and health that can be moderated by the role assumed by the leader. In addition, another study linked to these results establishes that moral values are highly related to the prosperity of workers; that is, a healthy work environment promotes wellbeing and it is that according to research, workers value working conditions to a great extent, even when there are positive and negative aspects, they remain firm in their work ethic in order to achieve greater wellbeing (King et al., 2011; Tacadena and Muico, 2022). Other studies that support the results of this research address work ethics as the construction of attitudes that derive from work-oriented values; in this way, a work environment governed by ethics and morality has a high potential to increase the general health of workers (Sakr et al., 2022; Zúñiga et al., 2022); in this context, it is highlighted that promoting ethical principles creates a work environment where employees feel valued and motivated, and that the connection between work ethics and general health contains a solid approach that fosters an ideal work environment for personal development and professional of an individual.

### Theoretical implications

This study leaves theoretical evidence of the connection of general health promoted as a result of servant leadership, brand love, and work ethic. The antecedents that were reviewed highlight that promoting servant leadership contributes to the general health of workers, so there is a need to create an adequate management culture in educational institutions, this implies maintaining a work environment where the leader promotes a climate of positive work that allows the wellbeing and health of workers. In addition, other research is highlighted that describes work ethics and love for the brand as a key element for the general health of workers, so a work environment where good practices exist will undoubtedly be a space where the worker express your wellbeing and general health, this being additionally supported by the emotional attachment you feel toward the institution where you work.

### Practical implications

A concrete measure to take into account in the study population is to train leaders with a focus on servant leadership, this through training programs for leaders in order to develop the necessary skills that allow them to efficiently guide the work group. Likewise, it is necessary to foster a work environment that promotes for the brand love, establishing strategies and policies that strengthen the sense of belonging and pride of workers toward their institution. Finally, it is important to also prioritize work ethics, for which clear policies must be established in favor of integrity, equity and mutual respect. Therefore, all actions together could significantly improve working conditions, generating a virtuous circle where workers feel valued, encouraging them to contribute to quality education and the achievement of common institutional objectives.

## Limitations and future research

Like previous studies, this research has some limitations that should be taken into account in future research. First, only one leadership style, servant leadership, was tested in this study. Therefore, future research should consider the use of other leadership styles (transformational, ethical, compassionate, charismatic, etc.) so that their behavior can be compared to a similar sample. Secondly, given that the study filtered out educational workers who had worked at the institution for a minimum of 6 months, it was a limitation for the research team, because in some institutions the worker was new staff, because the data began to be collected in the first 2 months of the academic year, in this sense, it is recommended to collect data after the half academic year, so that the leadership evaluation is relevant, unless an inclusion criterion unrelated to this is considered.

Third, the sociodemographic data from this study were not used in the hypothesis analysis. In future research, some sociodemographic data could be used as moderator variables, and consider expanding the sample. Fourth, this research only uses one leadership style, future research is expected to use charismatic leadership styles, transformational leadership, transactional leadership, business leadership, and e-leadership. And fifth, it is believed that it could be a great contribution to evaluate these constructs together with other associates, such as: environmental factors, job insecurity, labor ergonomics, among other important topics, as well as comparing the results in a longitudinal study.

And finally, based on the findings of this study, it is suggested that educational institutions promote the servant leadership style among their leaders and managers, including training in servant leadership skills and reinforcing programs that promote their application. In the same way, they should establish strategies that allow employees to foster love and loyalty toward their workplace, promoting the development of specialized programs that promote a healthy attachment to the brand. In addition to promoting a solid work ethic among leaders and employees, which allows favoring the organizational ethical climate. Knowing that this will also generate a better corporate reputation. In short, the results of this study have managed to achieve the proposed objectives and the hypotheses addressed at the beginning of the research have been tested, however, we believe that in future research the correlated construct could be evaluated in other economic sectors, as well as compare results over time.

## Conclusion

The present study demonstrated that the three constructs (servant leadership, brand love, and work ethic) predict the general health of workers in a positive and significant way, in a sample of Peruvian workers in the education sector. Furthermore, the results suggest that these factors can be used to improve the health of employees in educational institutions in Peru and possibly in other contexts as well. The nature of the factors has shown that servant leadership skills, rather than a theoretical definition, is a significant factor shaping the health and wellbeing of an educational business community. Better leadership fosters professional attitudes, encourages ethical behavior, and improves worker health.

## Data availability statement

The original contributions presented in this study are included in this article/supplementary material, further inquiries can be directed to the corresponding author.

## Ethics statement

The studies involving human participants were reviewed and approved by the Ethics Committee of the Universidad Peruana Unión (2022-CE-EPG-0000167). The participants provided their written informed consent to participate in this study.

## Author contributions

EL-A: Conceptualization, Data curation, Writing – original draft, Writing – review & editing. MV-G: Conceptualization, Investigation, Methodology, Project administration, Resources, Writing – original draft, Writing – review & editing. DM-L: Data curation, Formal analysis, Funding acquisition, Investigation, Resources, Software, Supervision, Validation, Visualization, Writing – review & editing.
